# One seasonal clock fits all?

**DOI:** 10.1007/s00359-023-01680-4

**Published:** 2023-11-10

**Authors:** Stephan Michel, Laura Kervezee

**Affiliations:** Department of Cell and Chemical Biology, Leiden University Medical Center, Postzone S5-P, 2300 RC, PO Box 9600, Leiden, The Netherlands

**Keywords:** Circadian clock, Photoperiod, Adaptation, Neuronal network, Comparative mechanisms

## Abstract

Adaptation of physiology and behavior to seasonal changes in the environment are for many organisms essential for survival. Most of our knowledge about the underlying mechanisms comes from research on photoperiodic regulation of reproduction in plants, insects and mammals. However, even humans, who mostly live in environments with minimal seasonal influences, show annual rhythms in physiology (e.g., immune activity, brain function), behavior (e.g., sleep–wake cycles) and disease prevalence (e.g., infectious diseases). As seasonal variations in environmental conditions may be drastically altered due to climate change, the understanding of the mechanisms underlying seasonal adaptation of physiology and behavior becomes even more relevant. While many species have developed specific solutions for dedicated tasks of photoperiodic regulation, we find a number of common principles and mechanisms when comparing insect and mammalian systems: (1) the circadian system contributes to photoperiodic regulation; (2) similar signaling molecules (VIP and PDF) are used for transferring information from the circadian system to the neuroendocrine system controlling the photoperiodic response; (3) the hormone melatonin participates in seasonal adaptation in insects as well as mammals; and (4) changes in photoperiod affect neurotransmitter function in both animal groups. The few examples of overlap elaborated in this perspective article, as well as the discussion on relevance for humans, should be seen as encouragement to unravel the machinery of seasonal adaptation in a multitude of organisms.

Raising questions is essential for scientific endeavors and discoveries and this format of a “perspective” may allow us to ask more questions than actually giving answers. To start with, what is a seasonal clock or is the term already misleading? As Colin Pittendrigh pointed out (Pittendrigh 1993), using powerful and persuading terms in science can also lead to asking the wrong question and a lot of frustrating research in the wrong direction. His example was the term “Zeitgedächtnis” (time memory) first introduced by Auguste Forel (Forel 1908) and then used by Ingeborg Beling, a co-worker of Karl von Frisch, to explain how bees know (remember) the time to fly out to a food source (Beling 1929). Instead of looking for a clock, the following research in bees was delayed for decades searching for a time memory.

So, when we say “clock” we may use the term that sells better, but we are studying a complex system of components with multiple levels from molecular to organs, even behavior. This is evident for the circadian clock, but may be even more important for the seasonal clock. Whatever form of seasonal adaptation we see in an organism, this is achieved by the coordinated work of multiple players including the circadian system. Two of the most applicable models originally explaining photoperiodic regulation in plants (Davis 2002) and insects (Saunders 2023; Pittendrigh 1972) suggest coincidence detection between either light and a circadian oscillation (external coincidence) or between two endogenous clocks (internal coincidence). While these models have been very helpful directing research, the exact mechanisms of photoperiodic time measurement and the role of circadian clocks still need more work. We find contributions of neuroendocrine systems, influence of light, temperature, and humidity; and we discover mechanisms at many levels of the organism that contribute to the measurement of day length, direction, and speed of change in photoperiod and to the adaptation of physiology and behavior (Takeda and Suzuki 2022; Hidalgo and Chiu 2023; Kostál 2006; Wood and Loudon 2014). It is not surprising that, depending on the task at hand (e.g., reproduction), the seasonal adaptation will require specific solutions in different organisms. A comprehensive review of all the excellent work performed on the mechanisms of seasonal adaptation in different organisms from plants to mammals would go far beyond the scope of this perspective piece. Therefore, we want to refer the reader to previous reviews on photoperiodism in plants (Gendron and Staiger 2023), insects (Takeda and Suzuki 2022) and mammals (Wood and Loudon 2014). The goal of this short perspective is to highlight some of the principles and mechanisms that show surprising similarities across species, even though we limit this to a few examples in mammals and insects. The title question is therefore meant as an encouragement to look for common mechanisms in photoperiodic regulation of different species, including humans.

## The question of seasonality

What seems like an almost trivial question of what defines seasonality can lead to controversy between researchers working on seasonal breeders and others working on melatonin-deficient mice. If one accepts that any adaptation of physiology or behavior of an organism to seasonal changes in environmental conditions represents seasonality, this trait may be viewed as a more general one and the search for underlying mechanisms broader. For instance, the elegant studies in insects not only reveal the neurohormonal pathways and mechanisms for photoperiodism warranting reproductive success in insects (Takeda and Suzuki 2022), but also describe the involvement with the circadian clock and even hint at some basic mechanisms that are also found in laboratory mice (see below). It goes without question that the seasonal adaptations in “real” animals require a fine-tuned system of elements, signals and pathways to detect or anticipate the change of seasons and prepare for either harsher conditions or get ready to mate and reproduce. However, using model systems deprived of, let us say melatonin, can help study and understand the underlying mechanisms at different levels of the photoperiodic encoding machinery (Meijer et al. 2010; VanderLeest et al. 2007). As an example, the seasonal modulation of daily locomotor activity patterns seems to be independent of melatonin (VanderLeest et al. 2007) and comparing these data with studies in melatonin-proficient mice can lead to further insights into the role of this hormone. On the other hand, working with melatonin-competent mice will pose other challenges like the question if the ‘neutral’ photoperiod of 12h light and 12h darkness is adequate for these animals or rather is moving their physiology into some unstable state flipping between long- and short-day behavior (Pfeffer et al. 2022). To add to the complexity, persistence of yearly changes in physiology and behavior in constant photoperiod has been observed in long-lived animals like sheep and birds, which has been termed circannual rhythms (Gwinner 2003; Lincoln et al. 2003). Finally, the question of how this all is applicable to humans may be the elephant in the room. But interestingly, as elaborated below, even though humans can cope and compensate for environmental changes, their physiology and behavior show clear seasonal modulation (Wehr 2001; Dopico et al. 2015) and its disruption can lead to serious health problems (Stevenson et al. 2015).

## Similarities between photoperiodic regulation in insects and mammals

Seasonal regulation of reproduction in insects has been well studied and photic as well as non-photic regulatory pathways have been described (Takeda and Suzuki 2022). Both involve the conversion of serotonin to melatonin as an initial step to break diapause, but the photoperiodic pathway uses the day length measure of the circadian clock, while the non-photic pathway is triggered by environmental factors such as temperature. Downstream, a number of species-dependent neuroendocrine pathways are involved, with pigment dispersing factor (PDF) as one common main factor conveying the photoperiodic information (Colizzi et al. 2023a, b; Hamanaka et al. 2023).

Like insects, mammals also use their central circadian clock, situated in the suprachiasmatic nucleus (SCN), to determine day length and convey this information by neurohormonal signaling to the neuroendocrine system mediating photoperiodic responses (Evans and Schwartz 2023). As in insects, the hormone melatonin also plays a central role in seasonal adaptation in mammals. The nightly release of melatonin from the pineal is controlled by the SCN (Illnerova 1991) and its daily profile reflects the change in day length throughout the seasons. Melatonin rise at the beginning of the night sets the phase of circadian rhythm of gene expression in the pars tuberalis (PT) and also suppresses a number of E-box controlled genes (Johnston et al. 2006). One of the genes rhythmically expressed and an important coactivator for thyroid-stimulating hormone (TSH) is eyes absent 3 (Eya3). With the phase of Eya3 rhythm set by melatonin onset and suppressed by the presence of melatonin, the coincidence between the light-dependent melatonin signal and the peak in Eya3 expression will trigger the seasonal response. Only in long days, the lack of melatonin allows for a sufficient peak in Eya3 expression to coactivate the expression of TSH, which in turn regulates the balance of thyroid-activating (type 2 deiodinase; DIO2) and -inactivating enzymes (DIO3) regulating the amount of the active form of thyroxine (T3) (Ono et al. 2008; Shinomiya et al. 2014). It is interesting that injection of melatonin into melatonin-deficient mice was able to lead to a similar ratio of DIO2/DIO3 as found in melatonin-proficient mice exposed to short photoperiod, suggesting that the downstream mechanisms are intact and comparable in both mouse models (Ono et al. 2008).

For the effect of melatonin on the PT, type 2 melatonin receptors (MT2R) in thyrotroph cells in the PT seem to be responsible. In comparison, the SCN expresses both MT1 and MT2 receptors and melatonin can elicit phase shifts of the SCN clock (McArthur et al. 1991). The interaction of melatonin and light at the level of SCN is not fully understood (Benloucif et al. 1999), but it has been suggested that activation of MT2R facilitates photic reentrainment in mice (Pfeffer et al. 2012), a mechanism that could explain the role of melatonin in controlling other seasonal behaviors (e.g., activity profile) as recently suggested (Pfeffer et al. 2022).

Another example of analogy to insect photoperiodism is the similarity between the signaling molecules PDF in insects and vasoactive intestinal peptide (VIP) in mammals (Vosko et al. 2007). PDF has been identified in many insects as the signaling molecule transmitting the time information from the central circadian clock cells (Renn et al. 1999; Helfrich-Förster et al. 2000; Beer et al. 2018; Petri and Stengl 1997; Singaravel et al. 2003). As mentioned above, PDF has also been found to be involved in photoperiodic responses in a number of insects (Hamanaka et al. 2023). A recent study showed a co-transcription factor EYES ABSENT (EYA) as the downstream target of PDF linking the circadian clock to photoperiodic regulation in *Drosophila* (Hidalgo et al. 2023). While the phase of rhythmic expression of Eya3 in the PT of mammals can be modulated by melatonin (Dardente et al. 2010; Masumoto et al. 2010), the mammalian analog of PDF—VIP—may also play a role in mediating circadian control of Eya3 expression. In mammals, VIP and its receptors have been extensively studied in the SCN and shown to be important for synchronization within the neuronal network of the SCN (Harmar et al. 2002; Colwell et al. 2003; Maywood et al. 2006; Ono et al. 2021). In addition, a study using in vivo SCN recording in freely moving mice suggested a role of VIP in seasonal encoding of day length in mice (Lucassen et al. 2012). Recently, it was also shown that short photoperiods increase the number of neuromedin-S (NMS) expressing neurons in the SCN and long photoperiod the number of VIP neurons (Porcu et al. 2022), suggesting a bidirectional relationship between photoperiod and neurotransmitters like VIP. In vitro studies proposed a synergistic interaction between VIP and GABA to regulate photoperiod-induced SCN network status of synchronization (Evans et al. 2013; Myung et al. 2015), but the mechanism how VIP is involved in photoperiodic regulation and the downstream signaling pathways still have to be investigated further.

A photoperiodic modulation of neurotransmitter function has been reported earlier in the SCN. The main neurotransmitter in the SCN is GABA, which can elicit either inhibitory or excitatory postsynaptic responses and therefore also determine an important neuronal network property, namely the balance between excitatory and inhibitory neuronal activity (E/I balance). Changes in photoperiod are associated with a modification of phase distribution of clock cells in the SCN (VanderLeest et al. 2007) and, at the same time, alterations in E/I balance (Farajnia et al. 2014; Myung et al. 2015). Mice exposed to long days will show a wider phase distribution of SCN clock cell neuronal activity and a higher E/I balance (more excitation and less inhibition) compared to mice exposed to short days. Since the polarity of the GABA response depends on the intracellular concentration of chloride ([Cl^−^]_i_), the co-transporters regulating [Cl^−^]_I_ were investigated in following studies using specific pharmacological blockers and recording molecular, cellular and behavioral outcome (Farajnia et al. 2014; Klett and Allen 2017; Olde Engberink et al. 2018; Rohr et al. 2019). The combined evidence suggests a critical role for the chloride co-transporter KCC2 (extruding Cl^−^ from the cell), the expression, and probably activity, of which in relation to its counterpart NKCC1 (bringing Cl^−^ into the cell) influences coupling and phase distribution of SCN cells in different photoperiods. Thus, the seasonal change in E/I balance is based on a change in GABA polarity, which is regulated by a modulation of KCC2. Interestingly, a recent study in Drosophila also showed the effect of KCC on GABA polarity in circadian pacemaker neurons and observed that downregulating KCC will change locomotor activity pattern in flies under long-day photoperiod (Eick et al. 2022).

## Seasonal adaptions in humans: a remnant of the past?

With the advent of electrical lighting, it seems likely that seasonal changes in light exposure and other environmental inputs have blurred in industrialized societies. Does this mean that humans are exempt from seasonal influences on their biology and that seasonality is merely a biological curiosity that can be studied in other species? The answer seems to be no. Firstly, in environments in which electrical lighting is pervasive, total light levels and its spectral composition humans are exposed to are still influenced by season, with higher illuminance during the afternoon and persisting longer into the evening during summer compared to winter (Thorne et al. 2009; Crowley et al. 2015; Shochat et al. 2019). Even in hospitals, seasonal changes in bedside light exposure have been observed (Van Der Linden et al. 2023), showing that seasonal influences may even be present in artificial environments where we least expect it—clearly depending on the architectural properties such as the presence and directionality of the windows. The obvious next question is whether seasonal changes in environmental cues such as light are sufficient to affect human physiology. It turns out that many bodily processes show time of year-dependent variation (Foster and Roenneberg 2008), ranging from sleep behavior (Kantermann et al. 2007; Mattingly et al. 2021), brain function (Zhang et al. 2023), and disease occurrence (e.g., affective disorders and infectious diseases) (Wirz-Justice 2018; Kronfeld-Schor et al. 2021) to tissue-specific gene expression (Wucher et al. 2023; Dopico et al. 2015), hormonal patterns (Tendler et al. 2021; Ikegami et al. 2019), and immune parameters (Wyse et al. 2021). With some exceptions (e.g., (Zerbini et al. 2021)), it should be noted that most of these seasonal changes have been observed at the population level, with most individuals contributing one or a few data points to the overall pattern. Hence, the magnitude of these changes within individuals is largely unknown, and, by extension, also the clinical relevance remains to be determined. Moreover, whether these seasonal variations continue to have adaptive value in industrialized societies or are remnants of an evolutionary past is equally unknown, but it is clear that seasonal changes in human physiology are pervasive.

Another question that is difficult to address in humans is how these seasonal changes in human physiology are brought about mechanistically. In other mammals, as discussed earlier (Evans and Schwartz 2023), it is the central circadian clock in the SCN that creates an internal representation of the duration of the day by adapting its daily electrical activity profile across the seasons. Simply put, the central clock regulates melatonin release by the pineal gland, thereby transmitting seasonal information to the rest of the body. In human post-mortem tissue, seasonal variation has been observed at the level of the SCN: the number of vasopressin-expressing neurons are highest in autumn and lowest in summer (Hofman and Swaab 1992). Interestingly, this variation is observed in young but not in elderly people (Hofman and Swaab 1994). However, whether there are seasonal changes in human melatonin rhythms or other outputs that could signal changes in photoperiod to other parts of the body remains unclear (Foster 2021). Indeed, studies that examined melatonin rhythms in humans in their habitual (electrically lit) environment have found minimal, if any, changes across the seasons (Stothard et al. 2017; Crowley et al. 2015), although these findings may depend on latitude (as discussed in (Wehr 2001)). In general, it seems that melatonin rhythms entrain to self-selected light exposure patterns that are influenced by local clock time, social demands, as well as natural (seasonal) changes (Zerbini et al. 2021). Therefore, it remains unknown what mechanism(s) drive the observed seasonal physiological changes in humans.

Another tantalizing, yet largely unexplored question, is to what extent individuals differ in their response to seasons. Given the large differences in light sensitivity of the circadian system that have been observed between individuals as measured by light-induced melatonin suppression (Phillips et al. 2019), it is plausible that there are considerable interindividual differences in the impact of season on physiology as well. This notion is corroborated by studies showing that seasonal responses are altered in individuals with seasonal affective disorder (SAD), for example with regard to melanopsin-driven pupillary light responses (Roecklein et al. 2021) and the duration of nocturnal melatonin secretion (Wehr 2001), which may explain differences in susceptibility to developing SAD.

## Conclusions

**Fig. 1:**
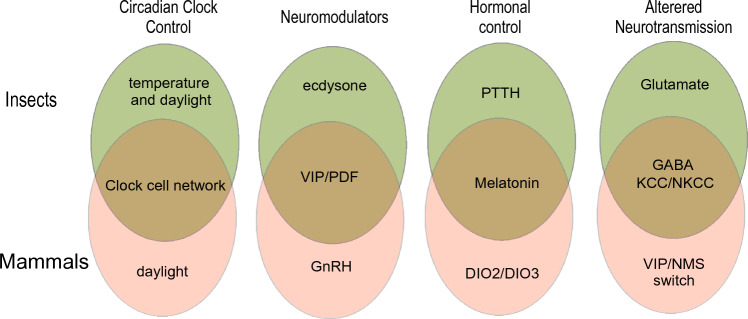
Examples of similarities (middle overlap) and differences (top and bottom) in principles or pathways/molecules involving photoperiodic adaptations in insects and mammals. *VIP* vasoactive intestinal protein, *PDF* pigment dispersing factor DIO2/DIO3: balance between type 2 deiodinase and type 3 deiodinase, *KCC/NKCC* potassium chloride exchanger/sodium potassium chloride exchanger, *NMS* neuromedin S, *GnRH* gonadotropin-releasing hormone, *PTTH* prothoracicotropic hormone

The answer to the provocative question in the title “one seasonal clock for all?” would be “not really”. Even within a single organism there are different solutions to regulate the timing of physiology (neurons in the central clock—peripheral clocks— red blood cells). The contribution of the circadian clock to seasonal adaptation is certainly different across the animal kingdom and depends on which behavior or physiological process is going to be controlled (e.g., reproduction or foraging). However, we keep discovering interesting overlaps between species in the principles of measuring day length, distributing this information and even the mechanisms employed (Fig. 1). Increasing evidence also suggests that humans should not lean back (“not my problem”), but in fact seem to have a functional seasonal clock that is only usually masked by our 24/7 society. In fact, seasonal influence is most obvious in changes of disease prevalence throughout the year. Moreover, seasonal changes drastically alter as climate change continues and may thus not only affect humans in their physiology, behavior and health, but, more importantly, will also affect the biosphere that we all live in and contribute to an increasing imbalance (Kronfeld-Schor et al. 2017). Therefore, we should recognize the relevance of clock-regulated seasonal adaptation even for humans and humbly accept the lessons we can all learn from a multitude of organisms.
